# Dual-hook clip closure for high-tension gastric defects

**DOI:** 10.1055/a-2897-7432

**Published:** 2026-07-14

**Authors:** Xiao Hu, Chenghong Li, Min Yang, Xinyu Huang, Yun-Chao Yang, Han Wang

**Affiliations:** 1 Department of Gastroenterology and Hepatology 89669 University of Electronic Science and Technology of China Sichuan Provincial People’s HospitalChengduSichuan ProvinceChina

**Fig. 1:**
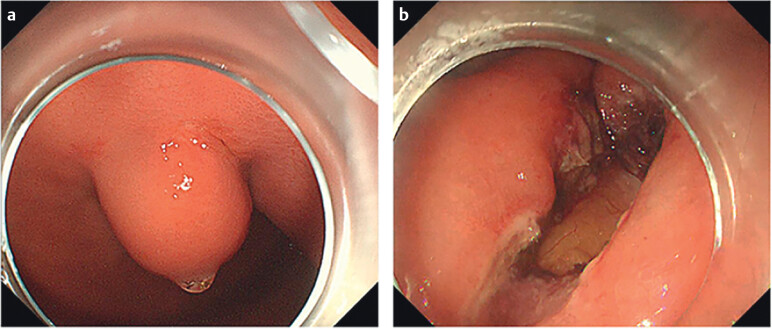
3.5-cm submucosal tumor located in the lower gastric body ( **a** ) and a full-thickness defect with inverted wound edges after endoscopic full-thickness resection ( **b** ).

**Fig. 2:**
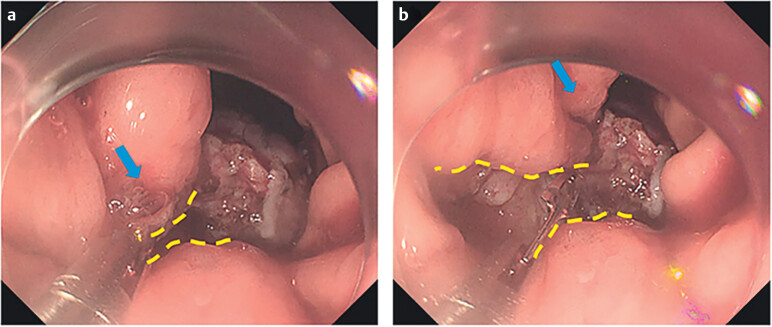
Traction-induced clip slippage with conventional clips. The blue arrow indicates the site of clip placement or slippage, and the yellow dashed line indicates the defect gap. Clipping of one mucosal edge with traction toward the contralateral side ( **a** ), followed by reopening of the defect gap after traction-induced clip slippage ( **b** ).

A 32-year-old woman presented with a 3.5-cm submucosal tumor in the lower gastric body ( Fig. 1a ). Endoscopic submucosal dissection was performed, and the lesion was found to originate from the muscularis propria. Full-thickness resection resulted in a large defect with high tension and inverted wound edges ( Fig. 1b ). Conventional through-the-scope clips repeatedly failed because tissue capture could not be maintained during traction ( Fig. 2 ). The complete procedure is shown in Video 1 .

**Video 1** Dual-hook clip closure using a hold-and-drag technique for a high-tension gastric defect after endoscopic submucosal dissection.


Traction-based closure techniques, such as the hold-and-drag method, have been introduced to facilitate approximation of opposing wound edges. 1​ ​2​ In addition, twin-clip systems have improved closure efficiency in large defects. 3​ However, these approaches may remain limited by insufficient tissue penetration, unstable anchoring in slippery or inverted mucosa, and reduced maneuverability in tangential defects.

**Fig. 3:**
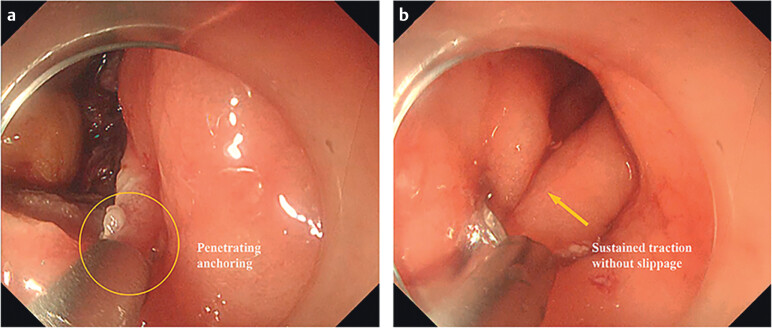
Stable traction with a dual-hook clip. Stable anchoring of one mucosal edge using deep hook engagement ( **a** ), followed by sustained traction with progressive reduction of the defect gap ( **b** ).

A dual-hook clip was therefore applied using a hold-and-drag technique. First, one edge of the mucosa was grasped using deep hook engagement to achieve stable anchoring and prevent slippage during traction ( Fig. 3a ). The tissue was then dragged toward the contralateral side to reduce the defect span. By opening the clip and adjusting its rotation, the opposite edge was engaged, followed by release to achieve initial approximation ( Fig. 3b ). The placement of the first dual-hook clip reduced wound tension, allowing subsequent closure with minimal traction force and eventual completion using standard clips.

Unlike edge-claw devices, the present device incorporates pin-like hooks and an off-tip hook configuration, thereby improving anchoring stability during traction in inverted defects and potentially reducing mucosal trauma during release.

**Fig. 4:**
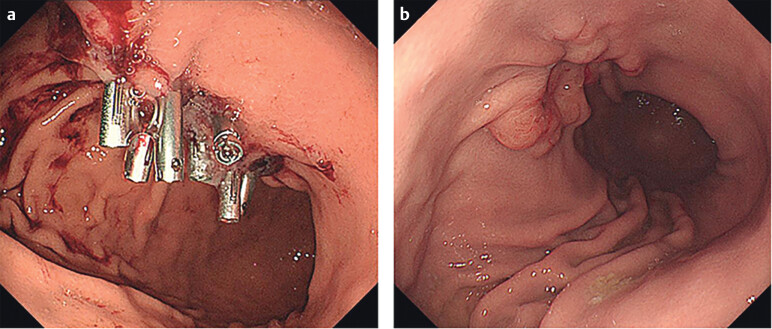
Successful closure and follow-up. Complete closure of the gastric defect ( **a** ) and healed scar at 3-month follow-up endoscopy ( **b** ).

**Fig. 5:**
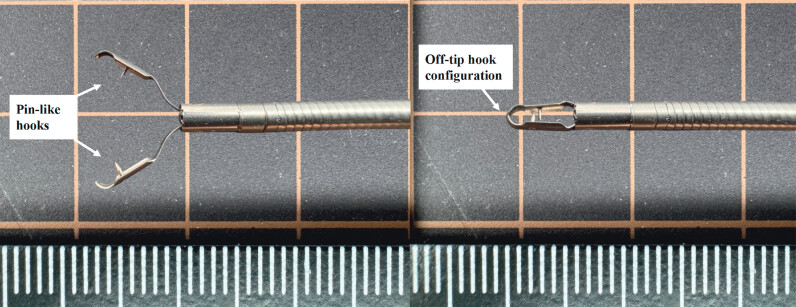
Design features of the dual-hook clip. Open state demonstrating the pin-like hooks, closed state demonstrating the off-tip hook configuration.

Complete closure was achieved, and adequate insufflation confirmed sealing integrity ( Fig. 4a ). Histopathological examination confirmed a gastrointestinal stromal tumor. At 3-month follow-up endoscopy, all dual-hook clips had spontaneously detached, and the defect had healed well ( Fig. 4b ). The design features of the dual-hook clip are illustrated ( Fig. 5 ). This technique appears reproducible and may facilitate the closure of large, high-tension, and full-thickness gastric defects.

Endoscopy_UCTN_Code_TTT_1AO_2AO
